# Factors impacting—stillbirth and neonatal death audit in Malawi: a qualitative study

**DOI:** 10.1186/s12913-022-08578-y

**Published:** 2022-09-22

**Authors:** Mtisunge Joshua Gondwe, Emily Joshua, Hendrina Kaliati, Mamuda Aminu, Stephen Allen, Nicola Desmond

**Affiliations:** 1 Department of Clinical Sciences, Liverpool School of Tropical Medicine, Pembroke Place, Liverpool, L3 5QA UK; 2 Behaviour and Health Group, Malawi Liverpool Wellcome Trust- Clinical Research Programme, PO Box 30096, Chichiri, Blantyre 3, Malawi; 3 Department of International Public Health, Liverpool School of Tropical Medicine, Pembroke Place, Liverpool, L3 5QA UK

**Keywords:** Stillbirths, Neonates, Death audit, Facilitators, Barriers, Health systems, Qualitative

## Abstract

**Background:**

Over one million babies are stillborn or die within the first 28 days of life each year due to preventable causes and poor-quality care in resource-constrained countries. Death audit may be a valuable tool for improving quality of care and decreasing mortality. However, challenges in implementing audit and their subsequent action plans have been reported, with few successfully implemented and sustained. This study aimed to identify factors that affect stillbirth and neonatal death audit at the facility level in the southern region of Malawi.

**Methods:**

Thirty-eight semi-structured interviews and seven focus group discussions with death audit committee members were conducted. Thematic analysis was guided by a conceptual framework applied deductively, combined with inductive line-by-line coding to identify additional emerging themes.

**Results:**

The factors that affected audit at individual, facility and national level were related to training, staff motivation, power dynamics and autonomy, audit organisation and data support. We found that factors were linked because they informed each other. Inadequate staff training was caused by a lack of financial allocation at the facility level and donor-driven approaches to training at the national level, with training taking place only with support from funders. Staff motivation was affected by the institutional norms of reliance on monetary incentives during meetings, gazetted at the national level so that audits happened only if such incentives were available. This overshadowed other benefits and non-monetary incentives which were not promoted at the facility level. Inadequate resources to support audit were informed by limited facility-level autonomy and decision-making powers which remained controlled at the national level despite decentralisation. Action plan implementation challenges after audit meetings resulted from inadequate support at the facility level and inadequate audit policy and guidelines at the national level. Poor documentation affected audit processes informed by inadequate supervision and promotion of data usage at both facility and national levels.

**Conclusions:**

Given that the factors that facilitate or inhibit audits are interconnected, implementers, policymakers and managers need to be aware that addressing barriers is likely to require a whole health systems approach targeting all system levels. This will require behavioural and complex intervention approaches.

**Supplementary Information:**

The online version contains supplementary material available at 10.1186/s12913-022-08578-y.

## Introduction

Preventable conditions cause more than 1 million stillbirths and neonatal deaths each year, with low- and lower-middle-income countries (LMICs) contributing more than 80% of these deaths [1, 2]. Despite increased facility-based birth, babies still die or develop lifelong disabilities after reaching facilities due to poor quality care [3–6]. Evidence suggests that death audit may be a valuable tool for improving quality of care, but only if the audit and feedback loop link to action at the point of care [7]. Despite the adoption of World Health Organization (WHO) stillbirth and neonatal death audit guidelines [8] by most LMICs, and the publication of quality improvement (QI) models [9–13], few countries have a fully functional stillbirth and neonatal death audit system. Furthermore, many audit action plans do not produce the desired change, with only a few being successfully implemented and sustained [14–16].

**Fig.1:**
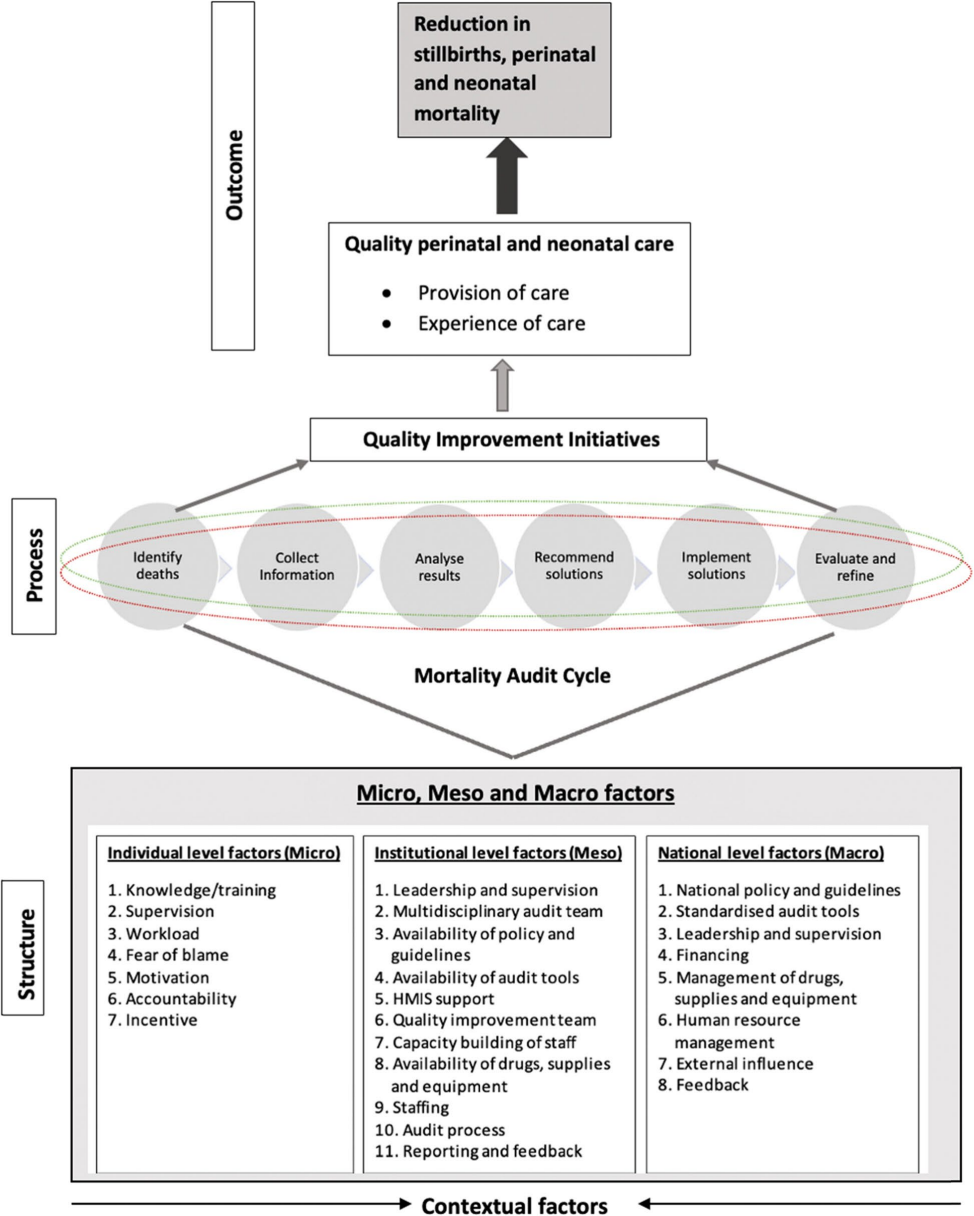
Conceptual framework/theory of change

The use of formal theories to inform strategies for implementing interventions to enhance benefits of death audits across settings has been advocated [17]. We reviewed several quality improvement models [9–13], to develop a conceptual framework to guide implementers, facility managers, policymakers and other stakeholders in understanding how structural factors are linked to the process of conducting stillbirth and neonatal death audit, which further link action plans to quality improvement initiatives (Fig. 1).

Whilst structural facilitators and barriers have been described in the literature [18, 19], addressing barriers to the success of the audit process in reducing mortality usually requires a change of the behaviour of multiple individuals and organizations. There has been little emphasis on the use of theories to identify behaviour change approaches to improve program implementation including death audit processes. Supporting staff to change their behaviour is key to successful intervention implementation [20]. This study explored factors that impacted staff participation in audit activities and the implementation of action plans with a view to developing a theoretically informed health system intervention.

## Methods

### Qualitative approach

This qualitative study was nested in a mixed methods research project evaluating stillbirth and neonatal death audit processes and neonatal outcomes in public hospitals in the southern region of Malawi. We used a conceptual framework to investigate into the key factors impacting audit processes at the individual, social and structural levels.

### Conceptual framework

Given the lack of explanatory frameworks relevant to factors affecting the conduct of death audit and implementation of audit action plans, we reviewed the literature and from this, developed and used a conceptual framework to guide this study (Fig. 1). The framework was developed from associations described in the literature between factors that affect the implementation of death audit at different levels [8–11, 13, 21, 22]. The conceptual framework informed the design and analysis of semi-structured interviews (SSIs) and focus group discussions (FGDs); guided the development of themes and helped align themes identified at individual (micro), facility (meso) or national (macro) level. Structure refers to characteristics of the setting in which audit is performed, process encompasses the components of the audit cycle and the interactions and outcome of audit [21].

### Study setting

The study was conducted in seven public hospitals from seven districts in the southern region of Malawi. The hospitals were purposively selected to provide a broad representation of health care workers involved in stillbirth or neonatal death audits and populations with a wide variation in district level neonatal mortality rates (see Appendix S1). Malawi's health system is organised at four levels (community, primary, secondary and tertiary) linked through an established referral system. Community, primary and secondary level care falls under district councils. The District Health Management Team (DHMT) is led by the Director of Health and Social Services (DHSS), who reports to the District Executive Committee locally and the central Ministry of Health (MoH). The selected hospitals included one tertiary hospital (hospital 1) and six secondary hospitals (hospitals 2–7). The tertiary hospital provides specialised inpatient and outpatient care at a regional level and receives referrals from district hospitals within the region and health centres within the district. The secondary (district) hospitals provide outpatient and inpatient services and receive referrals from community hospitals and health centres. All hospitals had a high patient load, hospital 2 had the highest frequency of audit meetings, hospitals 4 and 6 had very few audit meetings, and only hospitals 1 and 2 had DHMT members present during audit meetings [23].

### Researcher characteristics and reflexivity

The first author (MJG) conducted all SSIs and FGDs. With more than 14 years of experience as a nurse, MJG’s position as both a health professional and researcher balanced emic knowledge with an etic lens to deconstruct assumed knowledge and challenge where necessary [24]. Although MJG was known to some respondents prior to undertaking the study, the purpose of the interviews and her role was made clear to the participants and MJG was careful not to accept potentially common assumptions at face value. Furthermore, MJG kept reflexive diaries which enabled her to explicitly map her role as researcher, record and acknowledge her experiences, thoughts, opinions and feelings during data analysis and interpretation [25, 26]. The research team also had a field assistant (EJ) and a transcriber (HK), who were trained in qualitative research and assisted with note-taking during FGDs and transcription, respectively.

### Sampling strategy

We used purposive and convenience sampling to select respondents involved in the audit process. Purposive sampling enabled us to capture different experiences by age, cadre, ward, roles and years of experience, while convenience sampling was used to approach those staff with required categories available during the time of interview. We conducted SSIs and FGDs with nurses and clinicians involved in stillbirth and neonatal death audits and hospital/district management team members.

MJG approached potential respondents face-to-face for SSIs, provided them with information about the study's aims and secured written informed consent prior to arranging an interview for those who agreed to participate. Recruitment continued until the study team agreed to stop data collection due to data saturation when iterative analysis led to no further adjustments to the topic guides and no novel codes emerging [27]. Before each session, respondents' socio-demographic data was collected, including cadre, department, age, gender, level of education and years of experience. Interviews were carried out face- to -face in the respondent’s office or other private space. Only the participant and researcher (MJG) were present in the room during SSIs which lasted between 30 to 45 min. Respondents were able to use either English or the local language, Chichewa, at any point during interviews.

For FGDs, MJG provided information to audit committees and agreed on the discussion date and time. Only 1 FGD was conducted per hospital as the number of audit committee members ranged from five to 15 in each facility. FGDs lasted between 60 and 90 min.

Although there is a concern in the research that a group dynamic can undermine confidentiality and alter the depth of information provided [28–30], we incorporated pre-existing hierarchies (staff who already participate in death audit meetings) into the discussion. We also triangulated data collection methods by using both FGD and SSIs to mitigate the concerns.

### Data collection

SSIs and FGDs were conducted between July and December 2020. SSIs and FGDs were guided by semi-structured topic guides developed by the research team based on existing literature and conceptual framework domains (see Appendix S2). The topic guides explored experiences, facilitators and barriers in conducting stillbirth and neonatal death audit at the facilities and evolved following team discussions of emerging themes during the study period. FGDs also had an observer (EJ) who recorded non-verbal cues and kept time. MJG and EJ were trained in human subjects' procedures, confidentiality and privacy protection. All data were audio-recorded and transcribed verbatim in their original language by a professional transcriber (HK). To minimise losing meaning of the data, only the phrases from the native language transcripts that we would like to use as quotes were translated accurately to English. Interviews were anonymised through unique identifiers.

### Data management and analysis

The respondent demographic and interview data were stored in secured databases and computers accessible only to research staff with approved access. Using a thematic analysis guided by a framework approach, interview transcripts were analysed iteratively through a combined deductive and inductive approach using NVivo (V.12). Analysis was initially conducted deductively with predefined codes in the structural-contextual domain of the conceptual framework (Fig. 1). To reduce the potential bias and to ensure no important emerging themes were missed, MJG additionally conducted open coding on selected transcripts following familiarisation with the data by re-reading all the transcripts. After coding two interview transcripts, MJG, EJ and ND met to discuss the initial codes. MJG subsequently coded two more interview transcripts and an FGD transcript and built a coding tree inductively. After inductive coding of these five transcripts, MJG, EJ and ND reviewed the detailed codes and then considered these as they reflected the conceptual framework (Fig. 1) and predefined deductive codes under staff (micro), facility (meso) and national (macro) levels to ensure inclusion of all sub-themes within each overarching category. This final framework (codebook) was then used to code the remaining transcripts.

Initial themes were developed after coding all transcripts. MJG kept memos to mitigate her perspective and ensure her interpretation as a practising nurse was documented and accounted for and ran queries to identify patterns, similarities and differences in the identified themes across the facilities. These initial themes were then reviewed and refined according to the study's purpose and through the lens of the conceptual framework, which identified cross-cutting themes. Several team meeting discussions and reflections allowed continuous interaction with the data and a consensus to be reached where required.

#### Trustworthiness

Respondents were invited to review their transcripts, but only 20 respondents did. SSI and FGD data were triangulated to broaden the in-depth information from the interviews and compare across the facilities [31, 32]. Additionally, to provide data transparency, MG kept an audit trail by documenting all decisions made from conceptualisation through reporting [31, 32].

Ethical approval was obtained from the Malawi, College of Medicine (P.11/19/2869) and the Liverpool School of Tropical Medicine (19–076) ethics committees. All hospitals gave permission to conduct the study. All healthcare workers who agreed to participate in the study signed an ethics-approved informed consent form in English.

## Results

### Demographic characteristic of study participants

We interviewed 38 audit committee members individually of whom 22 (58%) were women and 5 (13%) were DHMT members, 22 (58%) were nurses and 11 (29%) were clinicians. Median (IQR) age was 34.5 (39–30) years and level of clinical experience ranged between 3 months and 30 years. There were 6 (16%) respondents each from hospitals 1,2, and 7 and 5 each (13%) from hospitals 3 to 6. Two (5%) respondents had a master's degree, 21 (55%) a degree and 15 (39%) a diploma. For nurses, 12 (55%) worked in nursery wards, six (27%) in labour wards and two (9%) each from postnatal and antenatal wards. For clinicians, six (55%) were allocated to maternity wards and five (46%) to nursery/paediatric wards. Eighteen (47%) SSI respondents also held significant roles such as programme coordinator or ward in charge. See Table S1 for a full description of each participant.

We also conducted 7 FGDs with a total of 49 respondents: 30 (61%) of whom were women, 9 (18%) were clinicians, and 40 (81.6%) were nurses working in nursery ward (20; 50.0%), labour ward (11; 28%), postnatal ward (5; 13%), nursery and paediatric wards (5; 13%), maternity ward (4; 10%) and two each (5%) from the antenatal and paediatric ward. Median (IQR) age was 32 (36–28) years and level of clinical experience ranged between 3 months and 27 years. Twenty-three (47%) had a degree while 26 (53%) had a diploma qualification. Similarly, to SSI respondents, 14 (29%) FGD respondents held other significant roles in their profession such as programme coordinator or ward in charge (see Table S2).

### Results overview

We identified 5 themes, which either facilitated or hindered conduct of audit meetings and the implementation of action plans. The identified themes are interrelated as they impact at both individual (micro), facility (meso) and national (macro) health system levels. Anything happening at individual level necessarily influences facility level practice and in turn, national level or district level actions inform capacity to implement at facility level. Table 1 summarises how the main themes crosscut at each level. We present these results according to the main themes that arose with some illustrative quotes from respondents. The main themes were training, staff motivation, power dynamics and autonomy, audit organisation and data support. In the following section, we will present these themes and show how they were interrelated across different levels within the health system.

**Table 1:** Emerging themes

	**Training**	**Staff motivation**	**Power dynamics and autonomy**	**Audit organisation**	**Data support**
**Individual level**	Inadequate training	Incentive	Inadequate resources	Meeting attendance restriction	Poor documentation
**Facility Level**	Lack of budget allocation for audit training	Leaders’ engagement	Lack of autonomy over procurement system	Attainment of a multidisciplinary team	Lack of data clerks
	Lack of peer-based training promotion		Lack of autonomy over recruitment system	Implementing action plans	Lack of data usage
**National level**	Donor support facility targeted training	Support from MoH	Decision making	Communication of audit findings	Mismatch of data indicators in the register
		Support from Donors			

### Training

This theme discusses compulsory staff training for those staff participating in audits. We report how inadequate training, lack of budget allocation for audit training, lack of peer-based training promotion and donor support for facility targeted training impacted training access and engagement amongst staff..

We found that where trainings were conducted, staff had adequate knowledge, understanding and acquired skills on audit. In contrast where it was not done, staff rarely engaged in audit activities. Trainings were done most frequently in hospital 2 as these were supported by an external donor, in contrast to hospitals 1 and 3 to 7 which had no external funding to promote audits. Staff from hospitals 1 and 3 -7 had only few staff trained as they waited for MoH initiated trainings on audit which was rarely conducted due to lack of funding. We also found that staff did not value in house training and peer-based training was not promoted at facility level due to the monetary incentive attached to externally funded training. DHMT rarely funded internal trainings due to inadequate funding and limited autonomy to make funding decisions and budgetary restrictions. Despite decentralisation, funding decisions are still taken at the national level. This led to dependency on external donors working in the district to fund trainings and resulted in inequity between hospitals.

'Attending training had helped me understand the importance of audit and I would more likely engage myself in audit’ (SSI, Safe Motherhood Coordinator, Hospital Matron, Hospital 3)

‘Most staff are new from pre-service. They needed training in death audit to be equipped with the necessary skills. But when we asked for funds from DHMT, they said funding was inadequate and they have no control as decisions were made at national level’ (FGD, Safe Motherhood Coordinator, Hospital 4)

'Three of us were trained and planning to train each other. However, staff wanted to attend external training themselves due to the attached monetary incentive’ (SSI, In-charge, Neonatal focal person, Hospital 3).

'Our partner (external donor) supports us with skills and training on how to conduct the death audits' (SSI, Clinician, Nursery ward, Hospital 2)

Individuals believe they do not receive adequate training as a result of facility-level decision-making and a lack of budget allocation, which is informed by a lack of clear national guidance on training approaches that can be used at facilities other than externally funded trainings. Because of decentralized decision-making and cost constraints, facility training is mostly driven by donors resulting in inconsistent access to training by staff across different facilities.

### Staff motivation

This theme explores the impact of staff motivation on implementing audit activities. Factors that affect staff motivation reported in this section include incentives, leaders’ engagement, support from MoH and support from donors.

Similarly, to training, staff attendance at audit meetings relies on incentives such as lunch allowance and refreshments. As such, Hospital 2, where these are consistently provided by an external donor, reported the greatest regularity in audit meetings.

Despite DHMT being aware of sustainability issues with monetary incentives, they rarely offered non-monetary incentives like supportive supervision, participation in audit meetings and recognition. But in the few hospitals (hospitals 1 to 3) where DHMT supported audits, respondents felt motivated, and most action plans were implemented. In hospitals 1 and 2 where the majority of audit meetings were conducted and management team members attended audit meetings, staff recognised the benefit of audit and staff were more committed and accountable than in other hospitals. Another non-monetary incentive respondents appreciated was MoH support in terms of quarterly supportive supervisions although these were rarely conducted. The unavailability of national level stillbirth and neonatal audit guidelines was a demotivator to staff performing audits. As such audits were not prioritised, rarely conducted, or valued at all levels. Other respondents emphasised the importance of publicizing newborn deaths in the same way that maternal deaths are publicized. This serves as a motivator for employees to undertake audits in order to ensure public accountability. In most hospitals frequency of audits was determined by the availability of external funding and internal funding from DHMT. Whilst donor support facilitated audits and staff motivation, DHMT was concerned with sustainability due to other funding priorities and an overall lack of resources.

‘If no monitory incentive, the members turn up would be poor, which left a lot of files unaudited thereby missing out some of the important action points’ (SSI, Clinician, Nursery, Hospital 2)

‘We needed more motivation from DHMT like appreciation, their attendance in audit meeting, refreshments or a bottle of water’ (FGD, Clinician, Hospital 6)

'With improvements seen in care, we were motivated to keep on doing audit meetings' (FGD, Nurse In-charge, Nursery ward, Hospital 2)

'The District Nursing Officer (DNO) and District Medical Officer (DMO) helped us mobilise resources, identified partners and organised mentorship when they attended audit meetings. Solutions were achieved quick and fast; the team was motivated’ (SSI, Neonatal focal person, Hospital 2)

‘…..to be honest on DHMT supervision, since I came here a year ago, we had never supervised the district hospital we usually went to the health centres' (SSI, District Medical Officer, DHMT member, Hospital 7)

‘The national level supervised us. Their feedback helped us improve the care and motivated us to continue doing audits as they asked for it during supervision’ (FGD, Paediatric Clinician, Hospital 3)

‘The maternal death audit has well-stipulated guidelines on when to audit and report to MoH, unlike neonatal death audits, I had not come across any guidelines’ (SSI, Deputy Safe Motherhood Coordinator, Labour Ward, Hospital 7)

'If a pregnant woman died, it became a public concern. Donors and management team would support it and staff attend it voluntarily’ (FGD, Clinician, Hospital 3)

‘The team requested our support in terms of finance or refreshments, but we could not sustain it, that was why we said no to financial incentive’ (SSI, District Nursing Officer, DHMT member, Hospital 2)

'….even sustaining audit meetings funding when partners pulled out it a challenge, we only fund occasionally' (SSI, District Medical Officer, Hospital 7)

The fact that individuals wait for availability of monetary incentives to convene or participate in audit activities is informed by facility level institutional norms of reliance on incentives and inadequate provision of non-monetary incentives, which in turn is informed by national level directives, policies and guidelines that allow donors to provide more monetary incentives than non-monetary and lack of national stillbirth and neonatal death audit policy and guidelines. The norm and culture of using monetary incentives to individuals when attending meetings or training dominate recognition of other audit benefits and provision of non-monetary incentives that have equal power to motivating staff. Nonetheless, facility managers' capacity to maintain monetary incentives is a concern.

### Power dynamics and autonomy

. This theme reports the impact of power dynamics at all system levels that affect resource management and audit implementation. The reported factors are lack of autonomy over procurement system, lack of autonomy over recruitment system and decision making.

Respondents were unable to implement audit activities in hospitals where resources were inadequate. The most inadequate resources included essential drugs, equipment and staffing. This was owing to centralized powers over resources, staffing and funding, which, notwithstanding decentralization, the facility level had no control over. With staff shortages and heavy clinical workloads, it was difficult to convene audit meetings or participate, which led to cancellations. Despite critical shortage of staff in wards, auditing all stillbirth and neonatal deaths was a requirement stipulated by the national-level authority. To fulfil the requirement, staff reported working in pairs to complete audits, as opposed to a multidisciplinary team approach and compromising the quality of audits. With more decision power at national level, DHMT members had limited autonomy and decision-making powers resulting in a disconnect between hospital needs and central level decisions. This led to competing priorities and inadequate or delayed funding which affected support of audit meetings, resources to implement audit solutions and staff training as described in the previous section.

‘We lacked supplies like oxygen, pulse oximeter, 50% dextrose, antibiotics and nasogastric tubes, which made us difficult to implement changes in care’ (SSI, Nurse/midwife Technician, Nursery ward, Hospital 7)

‘We plan, we implement, but we do not decide how much money would get, how many staff we would employ and what training our staff would attend. The national level had all powers to decide’ (SSI, District Medical Officer, DHMT member, Hospital 7)

‘We only have a sole medical supplier. We could not outsource, so the availability of drugs depended on those drugs at the central medical stores. We run out of options because of the policy and decision-makers sat at a national level’ (SSI, District Medical Officer, DHMT member, Hospital 7)

'Nurses and clinicians were few, but if we presented to DHMT, they said it was not in their capacity to decide but national-level’ (FGD, Paediatric Clinician, Hospital 3).

The situation that which staff (individuals) lacked resources to support stillbirth and neonatal death audit implementation is informed by limited facility-level autonomy and decision-making power created by national-level authorities, who are determinants for decision making at all system levels. Due to centralised decision-making powers, the facility level decisions are limited and mostly done at the national level which results in competing priorities and affects resource availability at facilities.

### Audit organisation

This theme reports gaps identified in areas of meeting attendance, attainment of a multidisciplinary team, implementing action plans and communicating audit findings.

Given that most audit meetings were done when there was funding in all hospitals, staff felt side-lined as most meetings were attended by invitation depending on budget restrictions as opposed to extended invitation in hospital 3. But even though the meeting invitation was open in hospital 3, it was difficult to get a full multidisciplinary team to attend audits due to busy schedules or no incentive, which led to meeting cancellations. Hospitals 1–4 resorted to having departmental audits which excluded staff from other disciplines. It was difficult to implement suggested actions due to inactive quality improvement teams with no link with the stillbirth and neonatal death audit in all hospitals However, despite some meetings being funded in hospitals 1,2,5, and 7, completing the WHO 6 audit cycle steps ( Fig.S1) was a challenge, with the last two steps rarely implemented as they were not included in the audit form template.

'I did not participate in audit meetings always. It depends if you were invited or not’ (SSI, Nurse, Nursery ward, Hospital 4).

‘We were supposed to have a hospital quality improvement team, but it is not active. There is a work improvement team available in the nursery, though inactive, and I did not even know who was in that committee’ (SSI, Paediatric Clinical Officer, Hospital 1).

'Mostly we met to do audits but not met to review previous action plans' (SSI, District Medical Officer, DHMT Member, Hospital 7)

‘…the form we used reports up to suggested solutions (action plan). No information on follow up of solutions or evaluation of process which limit staff in doing the steps’ (FGD, Paediatric Clinician, Hospital 1)

For all hospitals, even after the audit meetings, it was not clear where they report the audit findings as it was informed by the funding source rather than a well-defined line of authority. Although internal feedback was given in the wards, it lacked DHMT member representation. Since only two hospitals (hospital 1 and 2) conducted stillbirth audits separately, the majority of respondents claimed that stillbirths’ audits were undertaken concurrently with maternal deaths audits. Stillbirths were not prioritized during the process.

‘We only report to a funder, who funded the audit meeting, not to the national level (SSI, Neonatal focal person, Nursery ward, Hospital 5)

‘After the audits, we make two reports one for the district management through the DNO and the second one we report to NEST 360 which is our partner helping us in neonatal care which we gave a report that goes to the Ministry of Health’ (SSI, Nursery Incharge, Hospital 3)

‘When we give feedback to our junior colleagues, they do not take it seriously as we are on the same level. It would have been good if senior members were involved during ward or department feedback’ (FGD, Helping Babies Breath Coordinator, NMT, Labour ward, Hospital 7)

‘We have always started with maternal death audits, leaving no time for stillbirths, and we are mandated to report maternal deaths within 72 hours to Ministry of Health, which we do before stillbirths’ ( SSI, Deputy Safe motherhood Coordinator, Postnatal ward, Hospital 1)

The fact that the individuals were restricted to attend audit meetings, was attributed to an institution norm of reliance on monetary incentives, informed by national-level decisions on incentives as described in the previous section. It was also attributed to inadequate promotion of facilities to have active quality improvement teams due to inadequate enforcement at national level. It was also demoralising to individuals to see actions plans not implemented or reported, which was informed by inadequate support from DHMT as previously described, which in turn is informed by inadequate guidelines (audit form template) and lack of reporting system at national level. Well stipulated guidelines together with supportive supervision are more likely to motivate facility and staff in implementing audit activities.

### Data support

This theme reports data support challenges at all levels. The main challenges were poor documentation, lack of data clerks, lack of data usage and mismatch of data indicators in the register.

Incomplete patient information made analysis of death incomplete and affected audit outcomes across all facilities. Critical shortage of ward clerks in hospitals 2–7 made nurses responsible for entering data into the register. Timely data entry was difficult due to nurses’ busy schedules or inadequate ward clerks. Poor filing systems resulted in unavailability of the mothers’ clinical records which are key in analysing stillbirth and neonatal deaths. This was common in all hospitals, where most cases were referred from health centres, and where facilities waited a long time to audit deaths. Despite newborn data being collected, it was rarely used by staff during audit. One of the respondents pointed to incompatibility of data indicators between the ward register and the HMIS platform, which made its usage difficult.

‘Due to lack of adequate ward clerks, data entry was mostly incomplete. Sometimes nurses would help but because of their busy schedule, this was also impossible’ (FGD, Deputy Nurse Incharge, Nursery ward, Hospital 1).

‘Mostly the patient records were incomplete, maternal files, feeding charts were not attached, making analysis incomplete’ (SSI, Clinician, Nursery ward, Hospital 4)

‘We did not know how to use generated data. We waited for external assessors to use data for us, the same with audit data’ (FGD, Paediatric Clinician, hospital 3)

‘The HMIS list of diagnoses is not compatible with the diagnosis we made in the wards. In HIMS it was just neonatal complications and some of the diagnoses were not so descriptive that you could analyse’ (SSI, Deputy Head of Department, Paediatric ward, Hospital 1)

The fact that individuals did not complete patient information or attach appropriate forms, which affected audit process, was due to critical shortage of staff, inadequate guidelines and inappropriate guidance from facility level leaders, who rarely supervised staff. The decision-making powers at national level over staff recruitment contributed to ward clerk shortage in the facilities. Even though some data was collected, its usage by staff was not promoted at facility level and the newborn registers did not match with consolidated indicators in the HMIS platform which is controlled at the national level.

## Discussion

### Summary of the findings

We identified facilitators and barriers that affect staff engagement in audit activities including the implementation of action plans at all system levels. We found that the factors were interconnected, such that decisions made at national level informed decisions at facility level which in turn impacted staff behaviour at individual levels. Given that our original conceptual framework emphasised the role of different levels influencing the audit process, we have presented the results using this structure, highlighting the interconnections between each level. We have found primarily that the different levels inform and are informed by each other. The identified factors are related to training, staff motivation, power dynamics and autonomy, audit organisation and data support.

The facilitators at Individual level were adequate training, availability of financial incentives, and recognition of audit benefits while barriers were inadequate training, over-reliance on financial incentives, inadequate resources, meeting attendance restrictions and poor documentation.

At facility level, facilitators were availability of DHMT during audit meetings and barriers were lack of budget allocation for audit training, lack of peer-based training promotion, fears of sustaining financial incentives, inadequate non-monetary incentive, unavailability of DHMT members during audit meetings, lack of supportive supervision, limited autonomy and decision-making powers, inability to attain multidisciplinary team, inactive quality improvement team, shortage of staff, inability to complete the WHO audit cycle steps and lack of data utilisation.

While at national level, facilitators were training support, donor coordination and availability of national-level supportive supervision while barriers were donor policy, donor support sustainability, decision-making powers, lack of national-level stillbirth and neonatal death audit policy and guidelines, unstructured reporting and feedback system. In this section, we will discuss strategies that may help to capitalize on facilitators and reduce barriers in the following areas: training, incentives, leadership, donor support, power dynamics, decentralisation and guidelines.

#### Training

Trained staff with adequate skills and knowledge will likely be more effectively engaged in the audit process. Staff skills in the neonatal death audit process and knowledge of its aims, objectives and values are key to ensuring effective implementation. One of the identified facilitators was a supported training programme that equipped and motivated staff in hospital 2 to engage in audit activities. Similar findings have been reported in an integrative review on the impact of education and training interventions for nurses and other health care staff involved in the delivery of stroke care, where interactive education and training delivered to multi-disciplinary stroke teams were associated with a positive impact on patient and quality of care outcomes [33].

In facilities where supported training programmes were missing (hospitals 4 and 6) staff rarely engaged in audit and failed to recognise audit benefits on the provision of care. This finding is consistent with a study in Australia, where misinterpretation of intention and meaning of an intervention impacted staff engagement in the baby-friendly initiative [34]. Although these may clash with individual values, clearly stated values at the organisational-level influence staff's decisions about what you do and how you do it [35].

Inadequate knowledge, skills and understanding of the value of audit can be resolved through training [34, 36]. However, in this study, training was rarely conducted in hospitals 1 and 3–7 due to inadequate funding at facilities. In mitigation, there is a need for a structured plan on how knowledge or information should be transferred from trained staff to untrained staff. Furthermore, as staff expressed that they would like to attend external training due to the attached incentives, it was difficult for those already trained to transmit knowledge to untrained colleagues due to jealousy that they had received financial incentives while others covered their duties in the ward during the training period. But there was also inadequate promotion of peer-based training by management team members. Sensitisation of the value of implemented audit plans in improving health care is needed beyond personal benefits.

#### Incentives

While adequate skills are needed for staff to perform audit, skilled and motivated staff are more likely to engage in audit activities. Although monetary incentives were a facilitator and motivator for some staff to participate in audit, staff commitment was often low due to over-reliance on monetary incentives. This finding agrees with studies conducted in LMICs, that found it challenging to implement interventions with no monetary incentive [37–40]. While both monetary and non- monetary incentives determine staff motivation, managers in organisations spend less time and effort on non-monetary incentive measures [41]. This should be balanced in an organisation for staff to be motivated and be able to engage in activities.

Participants also mentioned barriers directly related to the hospital environment that demotivate them in engaging in audit process. These included resources, management support, inadequate staffing and busy ward schedules. Other studies have cited staff workload, shortages, staff turnover, changes in roster and lack of time for implementation as the most common barriers to audit [42–45]. System-level commitment and support from the management team are required to address barriers to audit [31, 32].

Staff may complain of busy schedules as an excuse for not attending audit meetings [46]. In this study, participants highlighted that using staff on duty to conduct audits created ward shortages. A study on stages of change for perinatal audit in South Africa suggested integration into a routine practice as one stage of change in audit, which could improve staff engagement to avoid perceiving audit as an external programme [47].

#### Leadership

Facility readiness to change organisational culture depends on leadership style, management orientation, accountability and human and material resource policies [48].

We found that leadership was limited in terms of supervision, recognition and DHMT participation in audit meetings which were missed opportunities for staff motivation. Stronger transformational leadership has been associated with positive work attitudes and high staff organisational commitment [49, 50].Transformational leadership is when leader's behaviours motivate and inspire people to perform beyond their perceived capabilities [51]. Transformational leadership also encourages followers to take moral responsibility for their actions, which is vital in auditing because the results of this study revealed a lack of staff and leadership accountability [52]. This sort of leadership is perfect for the audit process because it enables employees to recognize that change at the hospital occurs with and because of them rather than to them [52]. It also inspires followers to put their own interests aside for the good of the organisation [52]. We suggest that hospital management invests time and effort to use non-monetary incentives to motivate staff as an example of transformational leadership. Drawing on the WHO Health Systems Framework [53], which was incorporated in the study conceptual framework, it is also clear that the stillbirth and neonatal death audit programme should strive to reach a better balance between and among the six building blocks to achieve desired newborn health outcomes. We identified leadership and governance as a critical foundation, which could assist facilities in supporting other system blocks parameters during the implementation of audits.

#### Donor support

Given that donors and on a few occasions’ hospital management supported audit meetings with per diem or refreshments, staff were more willing to participate in audit meetings if such support were available as it motivated and supplemented their low wages. Similar findings have been reported in a study conducted in Malawi and Uganda that explored perceptions of per diems among government officers and non-governmental organization (NGO) officials, who reported that per diems provide benefits such as encouraging training, increasing staff motivation and supplementing salary [54]. Furthermore, another study done in Malawi also reported health workers scrambled for training if financial incentives were attached [37]. More adequate means to improve health workers' knowledge and motivation through supervision, onsite training and non-financial incentives are needed.

We observed donor dependency patterns in facilities where partners rather than the facilities themselves facilitated training, equipment supply and meeting allowances. Furthermore, donor dependency also encourages perception of audit as an external (externally funded and supported) initiative rather than a government driven requirement. According to the perspective of donors in a study conducted in Malawi, training was a quick fix to introduce new programmes or interventions. With number of trained staff as a key donor deliverable, they had no choice but to provide financial incentives for staff to attend training [37]. Although DHMT has the mandate to oppose donor-imposed training which is announced with short notice and limiting its inclusion in the district and MoH plans, it would be like standing in the way of their employers to receive financial incentives which supplement their low wages [37].

#### Power dynamics

Power dynamics and relationships play a part in intervention acceptability amongst staff, patients and management [55]. Participants identified management team members as key facilitators of audit implementation as they have the power of knowledge and decision making at the facility level. In hospitals where the management team supported audits through participation in audit meetings, staff were motivated. Furthermore, national-level supportive supervision in facilities facilitated staff engagement in audit activities. Similar findings have been reported in studies conducted in Pakistan and African countries, Ethiopia, Kenya, Malawi and Mozambique [56, 57].

#### Decentralisation

DHMT members also cited the difficulties resulting from the national level as the controllers of their human, material and funding resources. This finding agrees with a multi-country study where health system decision-making decentralisation is only on paper rather than in reality as the national level continues to control decision making for the district, in resources and staff hiring and dismissal [38]. Due to this, DHMT is disempowered and fails to act on district issues due to limited decision-making power, leading to inadequate resources and staff that affect how audit meetings and the implementation of action plans can be supported. We suggest that national and DHMT support is paramount in audit activities; their presence in audit meetings and support will likely facilitate and motivate staff in attending the audit and to ensure implementation of its action plans.

#### Guidelines

Guidelines from the national level act as an external influencer for staff motivation and engagement if perceived that they will benefit patient outcomes [34]. Despite WHO formulating and disseminating guidelines [8], implementation in LMICs is challenged by national-level factors. In an attempt to overcome these constraints, countries need to adapt the guidelines to suit the system and the context. Despite audit guidelines being adapted in Malawi, staff reported inability to complete the WHO audit cycle steps. Similar findings have been reported in a systematic review of LMICs [46]. Interventions implemented as part of hospital policy and translated into standard practice facilitate long-term change [58]. National guidelines for stillbirth and neonatal death audits, disseminated to facilities and training adequate staff would likely enhance staff capabilities to engage in neonatal death audit activities.

### Implications and potential interventions

The factors that affect the implementation of stillbirth and neonatal death audits are at all system levels and are interconnected. Addressing challenges at only one level is unlikely to be adequate but a comprehensive approach informed by behavioural theory that addresses factors at all system levels, recognising the relationships between levels, is more likely to be successful. I have also provided and evaluated the application of a conceptual framework/theory of change to guide implementers and policymakers to understand and optimise contextual factors affecting the success of stillbirth and neonatal audits.

The future intervention could be use of behaviour theory. Although the use of behaviour models is of great importance in QI processes and can assist in achieving success in audit implementation which is greatly impacted by staff behaviours. It is important to note that interventions focused only on the behaviour of staff are not likely to be successful without changes at other system level described in this study. The behaviour models have been rarely used in QI approaches but are widely used for health systems interventions and feature widely within the Medical Research Council (MRC) complex interventions framework and guidelines [59]*.* Several behaviour change models have been developed and used to understand behaviour, identify mechanisms of change, describe why programmes succeed or fail and guide in building better programmes [60]. But Michie, Atkins [61] have merged them into a workable and adaptable framework- the COM-B. The COM-B model proposes that people need capability (C), opportunity (O) and motivation (M) to perform a behaviour (B), which needs to be considered when designing an intervention [61].

Capability, opportunity and motivation interact to generate behaviour, which influences these components [62]. Changing one or more components of the behaviour system can alter staff engagement in audit activities [62]. Staff must have the appropriate knowledge and skills to perform audits and complete the audit cycle (capability), which can be acquired through mentorship and training. This skilled staff will require an opportunity to work in the environment in which he or she is employed, as well as the culture in which he or she is immersed. Audit policy and guidelines, resources, leadership involvement and data support are all possibilities. The staff's motivation to participate in audit activities might be influenced by both capabilities and opportunities. While this is a behaviour model, it also serves as a foundation for designing interventions to change behaviour [62]. Applying this to intervention design, the objective would be to determine what the behavioural target for employees to be able to engage in audit activities would be, as well as what aspects of the behaviour system would need to be changed to facilitate staff engagement in audit activities [62].

### Strengths and limitations

We used a conceptual framework developed from the quality improvement and health system strengthening models and applied it to describe barriers and facilitators to death audits. The framework has a solid theoretical base [8–11, 13, 21, 22], meaning that our findings are more likely to be generalisable and can be compared to similar programmes implemented in a similar context. We also used both SSIs and FGDs to explore the experiences of staff on stillbirth and neonatal death audits. This helped in consolidating both individual and group perspectives for comprehensive understanding of the concept. Furthermore, this is a multisite study that looks at the perspectives of workers from seven different locations and the data are compiled to present a full picture of the factors that influence audit implementation.

Our study had limitations because we only interviewed hospital workers and, due to time and financial constraints, we did not interview personnel from the Ministry of Health (national level) or external partners to get their perspective. Using purposive sampling to select sites and convenience sampling for study participants might have introduced sampling bias. However, triangulating data across the two collection methods ensured cadre level validation of key issues.

## Conclusions

We have identified multiple, interconnected factors that impacted audit implementation at individual, facility and national levels. The interventions required to promote facilitators and reduce barriers need to be comprehensive to address issues at all system levels. This will necessitate a combination of behavioural and complex intervention methods. Our findings will inform implementers, policymakers and managers to identify facilitators and address barriers to positively impact stillbirth and neonatal death audits and thereby improve the quality of neonatal care and outcomes.

## Supplementary Information

**Additional file 1.**

## Data Availability

The datasets used and analysed during the current study are available from the corresponding author on reasonable request. Most data generated or analysed during this study are included in this published article [and its supplementary information files].

## Abbreviations

COM-B: Capacity, Opportunity, Motivation and Behaviour
DHMT: District Health Management Team
DHSS: Director of Health and Social Services
DNO: District Nursing Officer
DMO: District Medical Officer
HBB: Helping Babies Breathe
FGDs: Focus group discussions
LMICs: Low-and lower-middle-income countries
MoH: Ministry of Health
QI: Quality Improvement;
SMC: Safe Motherhood Coordinator
SSIs: Semi-structured interviews
WHO: World Health Organization
