# Supplementary material for: Factors impacting—stillbirth and neonatal death audit in Malawi: a qualitative study
Source: BMC Health Serv Res. 2022 Sep 22;22:1191. doi: 10.1186/s12913-022-08578-y (PMC9502637; doi:10.1186/s12913-022-08578-y)
Supplement: Supplementary file 1 — Additional file 1. [file 12913_2022_8578_MOESM1_ESM.zip › Supplementary File/Appendix S2_Topic guides for SSIs and FGDs.docx]

**Appendix S2****: Topic Guides for SSIs and FGDs**

1. **Semi-Structured Interviews Topic Guide**

Start after self-introduction and getting the consent from the respondent.

1.Experience of conducting perinatal and neonatal mortality audit

(*Probe for more information on responsibilities, involvement in the perinatal/neonatal mortality audit committees in this hospital, for how long s/he has been involved in the committee*)

2. What do you think is working well in your facility regarding perinatal and neonatal death audit (*probe experience during audit process*)

3. What are/were the factors that facilitated implementation of perinatal and neonatal death audit in your facility?

4. What are/were some of the barriers/obstacles to the implementation of perinatal and neonatal death audit?

5. What changes would improve on the perinatal and neonatal death audit process in your facility?

6. What are your opinions on how audit is conducted and its impact on improving care (Probe on difference, if any, has it made to healthcare provider ability to provide or support care in maternity or neonatal unit?

7. Can you tell us about a time where the recommendations made during the mortality audit process resulted in a change in how care was provided?

8. Sometimes mortality audits can be a demoralising activity for staff. How is morale maintained in meetings?

9. In your view, how useful is perinatal and neonatal death audits for improving the quality of care and health outcomes for women and newborns in your facility?

1. **Focus Group Discussions Topic guide**

Start after self-introduction and getting the consent from the respondent.

1. How is the audit process conducted? (Probe for information on (who initiates the process of auditing? How soon after death is an audit conducted? How frequently are the audits conducted, who are involved in the typical case audit process (their professional and managerial positions), which cadre has the highest number? How the audit is done (map process using audit cycle) how are the deaths identified? Do you conduct audit on all deaths or only some? If only some, how is death selected for discussion? How is the information about perinatal/neonatal audit process collected and summarized? What materials are used for documenting the process, e.g., case notes, antenatal cards, partographs, delivery records, etc ? what trend or statistics data are routinely presented at audit? How are the solutions identified? How do you use the data on cause of death and modifiable factors to support solution identification? How does the mortality review team identify and prioritize recommendations? How are recommendations documented (specify areas captured)? How do you reach a consensus on recommendation, how long does an audit take to complete? Who analyses the results of an audit? What is the process for reporting back to the review team and other stakeholders on the status of recommendations (feedback system, institutional administration, partners and Ministry of Health as well as the community)?

2. What kind of support did you get from management members and other stakeholders during the process of audit and implementing change (service delivery, staffing, training, supervision, resources, health information system, finances, leadership and governance, QI team) the following people?

Ministry of Health (National)

Zonal level

District Health and Social Services DHSS/ Hospital Director

District Medical officer

District Nursing Officer/ Principal nursing Officer/matron

HMIS officer

Health services administrator

Sister in charge (neonatal unit or maternity)

Obstetrics (specialist/GMO)

Paediatric (specialist/GMO)

Quality assurance officer

Partners

Others

3. In your opinion, do the medical records and registers capture the necessary information for assessment of cause of death and contributing factors for maternal and perinatal deaths?

4.In your opinion, what are some facilitators and barriers to ensuring recommendations are implemented following mortality audit?

5. Describe the link if present between mortality audit information and any other quality improvement activities in your facility?

6. How are the audit recommendations used by the hospital managers and health policymakers in planning and budgeting to implement changes in the hospital? (Probe for more information about the use of the recommendations in planning, budgeting in order to implement changes in the hospital, examples of the changes that have ever been implemented as a result of recommendation of the audit committee since its establishment, who was involved in this change, how was the change implemented, what was the result of the change in the maternity or neonatal unit, reasons for failures).

7. Let us discuss the sustainability of this initiative (Perinatal and neonatal death review process). Probes: What factors will facilitate the sustainability of this initiative in your facility? Why and how will these factors help to sustain the initiative? What do you think are obstacles to the sustainability of this initiative in your facility? Why and how these obstacles will hinder the sustainability of mortality reviews?

8. Do you have any other suggestions to improve the audit mechanism in order to bring changes in obstetric and neonatal care in the hospital?
