# Supplementary material for: Factors impacting—stillbirth and neonatal death audit in Malawi: a qualitative study
Source: BMC Health Serv Res. 2022 Sep 22;22:1191. doi: 10.1186/s12913-022-08578-y (PMC9502637; doi:10.1186/s12913-022-08578-y)
Supplement: Supplementary file 1 — Additional file 1. [file 12913_2022_8578_MOESM1_ESM.zip › Supplementary File/Table S2_Characteristics of FGD participants.docx]

**Table S2: Characteristics of participants participated in seven focus group discussions (n=49)**

| **FGD Number** | **Facility name** | **Department** | **Cadre** | **Other roles** | **Age (years)** | **Gender** | **Level of education** | **Professional experience** |
| --- | --- | --- | --- | --- | --- | --- | --- | --- |
| **Focus Group Discussion 1** | |  |  |  |  |  |  |  |
| FGD 1.1 | Hospital 1 | Nursery/Paediatric | Clinical Officer | Neonatal Focal Person | 33 | M | Degree | 10 years |
| FGD 1.2 | Hospital 1 | Nursery ward | Nurse/Midwife Technician |  | 36 | F | College Diploma | 10years |
| FGD 1.3 | Hospital 1 | Nursery ward | Nurse/Midwife Technician |  | 26 | F | College Diploma | 1 year |
| FGD 1.4 | Hospital 1 | Nursery ward | Nursing Officer | Nursery ward in charge | 38 | F | Degree | 15years |
| FGD 1.5 | Hospital 1 | Nursery/Paediatric wards | Clinical technician |  | 36 | M | College Diploma | 10years |
| FGD 1.6 | Hospital 1 | Nursery ward | Nursing Officer | Deputy ward in charge | 32 | M | Degree | 9 years |
| FGD 1.7 | Hospital 1 | Nursery ward | Registered Nurse/Midwife |  | 42 | M | University Diploma | 10 years |
| **Focus Group Discussion 2** | |  |  |  |  |  |  |  |
| FGD 2.1 | Hospital 2 | Nursery ward | Nurse/Midwife Technician |  | 55 | F | College Diploma | 27years |
| FGD 2.2 | Hospital 2 | Maternity wards | Senior Medical Officer |  | 32 | M | Honours Degree | 3 months |
| FGD 2.3 | Hospital 2 | Nursery ward | Nursing Officer | Ward in charge | 35 | F | Degree | 12 years |
| FGD 2.4 | Hospital 2 | Nursery ward | Nurse/Midwife Technician |  | 28 | M | College Diploma | 3 years |
| FGD 2.5 | Hospital 2 | Nursery ward | Nurse/Midwife Technician |  | 38 | F | College Diploma | 10 years |
| FGD 2.6 | Hospital 2 | Nursery ward | Nurse/Midwife Technician |  | 50 | F | College Diploma | 26 years |
| FGD 2.7 | Hospital 2 | Labour ward | Registered Nurse/Midwife |  | 30 | F | University Diploma | 3 years |
| FGD 2.8 | Hospital 2 | Labour ward | Registered Nurse/Midwife |  | 26 | F | University Diploma | 1 year |
| **Focus Group Discussion 3** | |  |  |  |  |  |  |  |
| FGD 3.1 | Hospital 3 | Paediatric/Nursery ward | Clinical Officer |  | 40 | F | Degree | 14 years |
| FGD 3.2 | Hospital 3 | Nursery ward | Nursing Officer |  | 26 | F | Degree | 2 years |
| FGD 3.3 | Hospital 3 | Nursery ward | Nurse/Midwife Technician |  | 34 | F | College Diploma | 10 years |
| FGD 3.4 | Hospital 3 | Postnatal ward | Nurse/Midwife Technician |  | 23 | M | College Diploma | 2 years |
| FGD 3.5 | Hospital 3 | Paediatric ward | Nursing Officer | Paediatric ward In charge | 32 | F | Degree | 8 years |
| FGD 3.6 | Hospital 3 | Labour ward | Nursing Officer | Labour ward in charge | 38 | M | Degree | 16 years |
| FGD 3.7 | Hospital 3 | Paediatric/Nursery wards | Paediatric Clinical Officer |  | 35 | M | Degree | 8 years |
| **Focus Group Discussion 4** | |  |  |  |  |  |  |  |
| FGD 4.1 | Hospital 4 | Labour ward | Nursing Officer |  | 29 | F | Degree | 4 years |
| FGD 4.2 | Hospital 4 | Nursery ward | Nursing Officer |  | 28 | F | Degree | 2 years |
| FGD 4.3 | Hospital 4 | Postnatal ward | Nursing Officer |  | 27 | M | Degree | 2 years |
| FGD 4.4 | Hospital 4 | Paediatric ward | Nursing Officer |  | 28 | M | Degree | 2 years |
| FGD 4.5 | Hospital 4 | Labour ward | Nursing Officer | Safe Motherhood Coordinator | 32 | F | Degree | 8 years |
| **Focus Group Discussion 5** | |  |  |  |  |  |  |  |
| FGD 5.1 | Hospital 5 | Labour ward | Nursing Officer | Labour ward in charge | 32 | F | Degree | 8 years |
| FGD 5.2 | Hospital 5 | Nursery ward | Nursing Officer | Neonatal focal person/Nursery in charge | 30 | F | Degree | 6 years |
| FGD 5.3 | Hospital 5 | Nursery ward | Nurse/Midwife Technician |  | 29 | M | College Diploma | 5 years |
| FGD 5.4 | Hospital 5 | Postnatal ward | Nursing Officer |  | 22 | F | Degree | 3 months |
| FGD 5.5 | Hospital 5 | Maternity wards | Clinical technician |  | 26 | F | Diploma | 3 years |
| FGD 5.6 | Hospital 5 | Postnatal ward | Nurse/Midwife Technician |  | 40 | F | Diploma | 1 year |
| FGD 5.7 | Hospital 5 | Paediatric/Nursery wards | Clinical Officer |  | 30 | M | Degree | 6years |
| FGD 5.8 | Hospital 5 | Nursery ward | Nursing Officer |  | 23 | F | Degree | 1 year |
| FGD 5.9 | Hospital 5 | Postnatal ward | Nurse/Midwife Technician |  | 26 | F | College Diploma | 2 years |
| FGD 5.10 | Hospital 5 | Labour ward | Nurse/Midwife Technician |  | 34 | F | College Diploma | 5 years |
| **Focus Group Discussion 6** | |  |  |  |  |  |  |  |
| FGD 6.1 | Hospital 6 | Nursery ward | Nurse/Midwife Technician |  | 28 | F | College Diploma | 6 years |
| FGD 6.2 | Hospital 6 | Antenatal ward | Nurse/Midwife Technician | Safe Motherhood Coordinator | 28 | M | College Diploma | 4 years |
| FGD 6.3 | Hospital 6 | Labour ward | Nursing Officer | Labour ward in charge | 30 | F | Degree | 3 years |
| FGD 6.4 | Hospital 6 | Nursery ward | Nursing Officer | Nursery ward in charge | 23 | F | Degree | 1 year |
| FGD 6.5 | Hospital 6 | Labour ward | Nurse/Midwife Technician |  | 26 | M | College Diploma | 4 years |
| FGD 6.6 | Hospital 6 | Maternity ward | Clinical technician |  | 28 | M | Diploma | 3 years |
| **Focus Group Discussion 7** | |  |  |  |  |  |  |  |
| FGD 7.1 | Hospital 7 | Antenatal ward | Registered Nurse/Midwife |  | 35 | F | University Diploma | 10 years |
| FGD 7.2 | Hospital 7 | Nursery ward | Nurse/Midwife Technician |  | 38 | F | College Diploma | 8 years |
| FGD 7.3 | Hospital 7 | Labour ward | Nurse/Midwife Technician | Helping Baby Breathe (HBB) coordinator | 40 | F | College Diploma | 15 years |
| FGD 7.4 | Hospital 7 | Labour ward | Nursing Officer | Deputy Safe Motherhood Coordinator | 34 | M | Degree | 11 years |
| FGD 7.5 | Hospital 7 | Maternity wards | Clinical technician |  | 37 | M | Diploma | 2 years |
| FGD 7.6 | Hospital 7 | Nursery ward | Nurse/Midwife Technician |  | 30 | M | College Diploma | 6 years |
