# Supplementary material for: Factors impacting—stillbirth and neonatal death audit in Malawi: a qualitative study
Source: BMC Health Serv Res. 2022 Sep 22;22:1191. doi: 10.1186/s12913-022-08578-y (PMC9502637; doi:10.1186/s12913-022-08578-y)
Supplement: Supplementary file 1 — Additional file 1. [file 12913_2022_8578_MOESM1_ESM.zip › Supplementary File/Appendix S1_Criteria for hospital selection.docx]

**Appendix S1:** **Criteria for hospital selection**

1. A government public hospital
2. Either central or district hospital
3. Located in southern region of Malawi
4. Hospitals that conduct regular stillbirth or neonatal death audit
5. Wide range of neonatal mortality rates by including lowest, medium and highest district neonatal mortality rates (15-30 per 1000 births)
