# Supplementary material for: Factors impacting—stillbirth and neonatal death audit in Malawi: a qualitative study
Source: BMC Health Serv Res. 2022 Sep 22;22:1191. doi: 10.1186/s12913-022-08578-y (PMC9502637; doi:10.1186/s12913-022-08578-y)
Supplement: Supplementary file 1 — Additional file 1. [file 12913_2022_8578_MOESM1_ESM.zip › Supplementary File/Table S1_Characteristics of SSI participants.docx]

**Table S1: Characteristics of participants participated in semi-structured interviews (n=38)**

| **participant ID** | **Facility name** | **Department** | **Cadre** | **Other roles** | **Age (years)** | **Gender** | **Level of education** | **Professional experience** |
| --- | --- | --- | --- | --- | --- | --- | --- | --- |
| 1 | Hospital 1 | Nursery ward | Nursing Officer | Neonatal focal person | 29 | F | Degree | 4 years |
| 2 | Hospital 1 | Nursery ward | Nurse/Midwife Technician |  | 36 | F | College Diploma | 10years |
| 3 | Hospital 1 | Nursery ward | Nursing Officer | Nursery ward In charge | 38 | F | Degree | 15years |
| 4 | Hospital 1 | Nursery/Paediatric wards | Clinical technician |  | 36 | M | College Diploma | 10years |
| 5 | Hospital 1 | Postnatal ward | Nursing Officer | Safe Motherhood Coordinator | 30 | F | Degree | 5years |
| 6 | Hospital 1 | Management team member | Chief Clinical Officer | Deputy Head of Department | 51 | M | College Diploma | 29 years |
| 7 | Hospital 2 | Labour ward | Senior Nursing Officer | Safe Motherhood Coordinator/ hospital Matron | 30 | F | Degree | 5years |
| 8 | Hospital 2 | Maternity wards | Senior Medical Officer |  | 32 | M | Honours Degree | 3 months |
| 9 | Hospital 2 | paediatric/Nursery wards | Clinical technician |  | 41 | M | College Diploma | 6 years |
| 10 | Hospital 2 | Nursery ward | Nurse/Midwife Technician |  | 55 | F | College Diploma | 27years |
| 11 | Hospital 2 | Nursery ward | Nurse/Midwife Technician | Neonatal focal person | 39 | F | College Diploma | 17 years |
| 12 | Hospital 2 | Management team member | District Nursing Officer |  | 55 | F | Degree | 30years |
| 13 | Hospital 3 | Maternity wards | Senior Nursing Officer | Safe Motherhood Coordinator | 37 | M | Master’s Degree | 12 years |
| 14 | Hospital 3 | Paediatric/Nursery wards | Paediatric Clinical Officer |  | 35 | M | Degree | 8 years |
| 15 | Hospital 3 | Nursery ward | Nursing Officer |  | 26 | F | Degree | 2 years |
| 16 | Hospital 3 | Maternity and general wards | Chief Clinical Officer |  | 50 | M | Degree | 30 years |
| 17 | Hospital 3 | Nursery ward | Nursing Officer | Neonatal focal person/ In charge | 33 | F | Degree | 10 years |
| 18 | Hospital 4 | Management team member | Acting Administrator | Transport Officer | 58 | M | Diploma | 30 years |
| 19 | Hospital 4 | Nursery ward | Nurse/Midwife Technician |  | 57 | F | College Diploma | 27 years |
| 20 | Hospital 4 | Labour ward | Nursing Officer | Safe Motherhood Coordinator | 32 | F | Degree | 8 years |
| 21 | Hospital 4 | Nursery ward | Nursing Officer | Neonatal Focal Person/Nursery in charge | 30 | F | Degree | 6 years |
| 22 | Hospital 4 | Maternity and general wards | Senior Medical Officer |  | 25 | M | Honours Degree | 5 months |
| 23 | Hospital 5 | Labour ward | Nursing Officer | Labour ward in charge | 32 | F | Degree | 8 years |
| 24 | Hospital 5 | Postnatal ward | Nurse/Midwife Technician |  | 26 | M | College Diploma | 4 years |
| 25 | Hospital 5 | Nursery ward | Nursing Officer | Neonatal focal person/ in charge | 30 | F | Degree | 6 years |
| 26 | Hospital 5 | Paediatric/Nursery wards | Clinical Officer |  | 30 | M | Degree | 6years |
| 27 | Hospital 5 | Management team member | District Nursing Officer |  | 53 | F | Degree | 32 years |
| 28 | Hospital 6 | Management team member | Hospital Matron |  | 38 | F | Master’s Degree | 8 years |
| 29 | Hospital 6 | Maternity ward | Clinical technician |  | 28 | M | Diploma | 3 years |
| 30 | Hospital 6 | Nursery ward | Nursing Officer | Nursery ward in charge | 23 | F | Degree | 1 year |
| 31 | Hospital 6 | Antenatal ward | Nurse/Midwife Technician | Safe Motherhood Coordinator | 28 | M | College Diploma | 4 years |
| 32 | Hospital 6 | Labour ward | Nursing Officer | Labour ward in charge | 30 | F | Degree | 3 years |
| 33 | Hospital 7 | Labour ward | Nursing Officer | Safe Motherhood Coordinator | 34 | M | Degree | 11 years |
| 34 | Hospital 7 | Antenatal ward | Registered Nurse/Midwife |  | 35 | F | University Diploma | 10 years |
| 35 | Hospital 7 | Maternity wards | Clinical technician |  | 37 | M | Diploma | 2 years |
| 36 | Hospital 7 | Nursery ward | Nurse/Midwife Technician |  | 38 | F | College Diploma | 8 years |
| 37 | Hospital 7 | Labour ward | Nurse/Midwife Technician | Helping Baby Breathe (HBB) coordinator | 40 | F | College Diploma | 15 years |
| 38 | Hospital 7 | Management team member | District Medical Officer |  | 30 | M | Honours Degree | 3 years |
