# Supplementary figures and images for: Factors impacting—stillbirth and neonatal death audit in Malawi: a qualitative study
Source: BMC Health Serv Res. 2022 Sep 22;22:1191. doi: 10.1186/s12913-022-08578-y (PMC9502637; doi:10.1186/s12913-022-08578-y)

**Fig.S1: Mortality Audit Cycle**


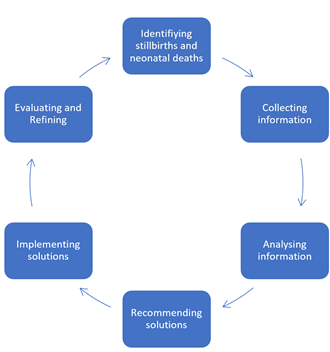


Source: Adapted from [World Health Organization (2016)](#_ENREF_209)

Supplement: Supplementary file 1 — Additional file 1. [file 12913_2022_8578_MOESM1_ESM.zip › Supplementary File/Fig S1_Mortality audit cycle.docx]
